# Neuro-Ophthalmic Manifestation Associated With COVID-19 in a Tertiary Eye Center in Nepal

**DOI:** 10.1155/crop/6694537

**Published:** 2025-07-26

**Authors:** Sanjeeta Sitaula, Chiranjiwi Shah, Ganga Sagar Shah, Rajeev Ojha

**Affiliations:** ^1^Department of Ophthalmology, B. P. Koirala Lions Centre for Ophthalmic Studies, Institute of Medicine, Tribhuvan University, Kathmandu, Nepal; ^2^Department of Optometry, B. P. Smriti Hospital, Kathmandu, Nepal; ^3^Department of Ophthalmology, Biratnagar Eye Hospital, Biratnagar, Nepal; ^4^Department of Neurology, Tribhuvan University Teaching Hospital, Institute of Medicine, Tribhuvan University, Kathmandu, Nepal

**Keywords:** cerebral venous sinus thrombosis, COVID-19, lateral rectus palsy, neuro-ophthalmic manifestation, nonarteritic ischemic optic neuropathy

## Abstract

The coronavirus disease 2019 (COVID-19) primarily involves the respiratory system, but can manifest with a variety of neuro-ophthalmic symptoms. Here, we describe three cases presenting with neuro-ophthalmic manifestations secondary to COVID-19 at a tertiary center in Nepal. The first case was a 42-year-old male with sudden onset painless loss of vision noticed in the right eye (RE) after COVID-19 infection. Examination findings in the RE showed best corrected visual acuity (BCVA) of 6/18 with relative afferent pupillary defect positive and superior sectoral disk edema in the same eye. The case was diagnosed as RE nonarteritic ischemic optic neuropathy associated with COVID-19 infection. Our second case was a 41-year-old female who developed bilateral sudden diminution of vision associated with headache and vomiting on the third day of testing positive for COVID-19 infection. She had bilateral BCVA of 6/12 and sluggishly reacting pupils in both eyes. Dilated fundus examination showed established disk edema. Imaging of the brain showed dural venous sinus (transverse and sagittal) thrombosis. So, the diagnosis of papilledema secondary to COVID-19 associated cerebral venous sinus thrombosis (CVST) was established. The third case was a 40-year-old male with right sixth cranial nerve palsy, ischemic stroke involving the right occipital lobe and posterior limb of the right internal capsule along with thrombosis of the left common iliac artery in the absence of any other preexisting vascular risk factors. Severe inflammatory reaction to COVID-19 causing a hypercoagulable state may be the causal factor in neuro-ophthalmic findings in our case series.

## 1. Introduction

The novel coronavirus disease 2019 (COVID-19), caused by severe acute respiratory syndrome coronavirus 2 (SARS-CoV-2), has caused a global pandemic in the past few years. Although respiratory disease is the most common manifestation of COVID-19, the virus can affect multiple organs, including the eye and the nervous system. Coronavirus comprises a family of enveloped, positive-sense, single-stranded, RNA viruses [1]. Its infection leads to a severe inflammatory response with hypercoagulability, thrombosis, and hypoxemia causing various pulmonary and extrapulmonary disorders [2]. It can also manifest with a variety of afferent and efferent neuro-ophthalmic disorders as recognized by its neurotropic and neuroinvasive capabilities [3, 4]. More than a third of patients with COVID-19 have neurological complications [5, 6], and the incidence appears to be higher in patients with more severe infections [7]. The principal neuro-ophthalmic manifestations reported include nonarteritic ischemic optic neuropathy (NAION) [8, 9], optic neuritis [10, 11], cranial neuropathies [12, 13], Miller–Fischer syndrome [13, 14], and myasthenia gravis [15]. Here, we describe three cases presenting with neuro-ophthalmic manifestations secondary to COVID-19 at a tertiary center in Nepal. This report adheres to the tenets of the Declaration of Helsinki. Written informed consent was obtained for use of clinical information and photographs for educational purposes. The requirement for ethical approval was waived by the institutional review board as this case series involved only three cases.

## 2. Case Reports

### 2.1. Case 1

**Figure 1:**
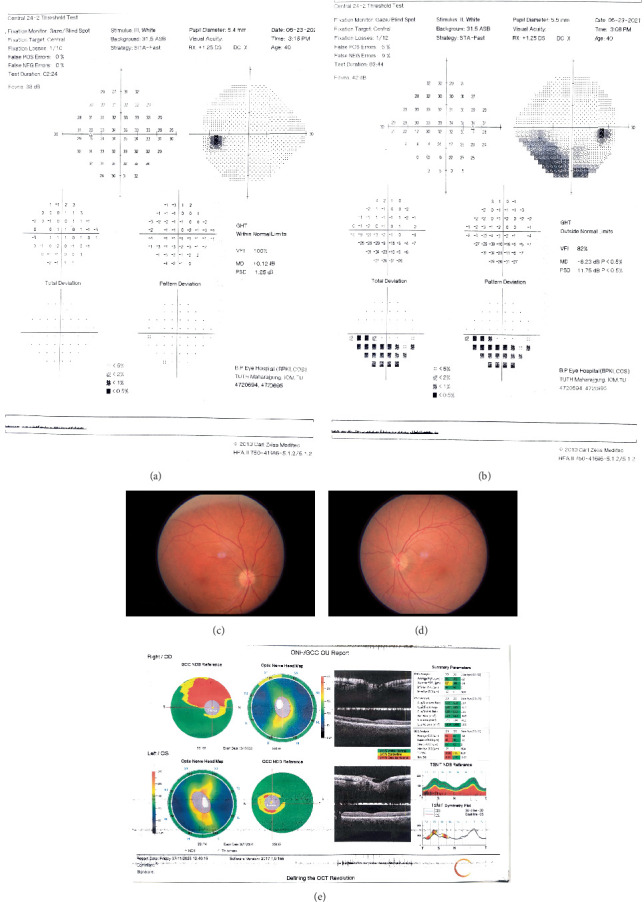
Humphrey visual field analysis [24–2] of (a) the left eye showing a normal pattern and (b) the right eye showing an inferior altitudinal field defect. (c, d) Color fundus photograph of the right and left eyes. The right eye shows a superior sectoral optic disk pallor, whereas the left optic nerve shows normal color of the optic disk with a small cup-to-disk ratio (anatomically) crowded disk. (e) Retinal nerve fiber layer (RNFL) analysis by SD-OCT (Optovue) showing superior RNFL thinning in the right eye measuring 81 *μ*m versus 105 *μ*m in the left eye due to superior sectoral optic atrophy. Macular ganglion cell–inner plexiform layer complex (GCC) analysis shows thinning of the superior GCC in the left eye (68 *μ*m) when compared to the superior GCC of the right eye (90 *μ*m).

A 42-year-old male presented with sudden onset painless loss of vision noticed in the right eye (RE) on awakening in the morning since the last 7 days following a diffuse, dull aching type of headache not associated with nausea or vomiting. He had tested positive for COVID-19 3 weeks ago after having mild upper respiratory tract symptoms, which were treated with home isolation and symptomatic treatment. He had no systemic comorbidities or vasculopathic risk factors. On examination, he had the best corrected visual acuity (BCVA) of 6/18 in the RE and 6/6 in the left eye (LE) for distance. Examination of the pupil revealed Grade I relative afferent pupillary defect (RAPD) in the RE. Other anterior segment examinations were normal in both eyes. Dilated fundus examination showed superior sectoral disk edema in the RE and a crowded optic disk (disk at risk) in the LE. He had total color blindness in both eyes and had a contrast sensitivity of 1.85 and 1.95 log units in the RE and LEs, respectively, as tested on the Pelli–Robson Chart. His RE had an inferior altitudinal visual field defect, whereas the LE had a normal visual field in automated visual field (AVF) 24-2 examination, as shown in Figure 1a,b.

Blood investigations for HbA1c, liver function test, renal function test, thyroid function test, lipid profile, erythrocyte sedimentation rate (ESR), and C-reactive protein (CRP) were within the normal range (Table 1). Other blood investigations like ANA, RA, VDRL, serology for HIV and HBsAg, and PT/INR were also negative. Furthermore, hypercoagulability workup was also negative and his sleep study was unremarkable. Magnetic resonance imaging (MRI) of the brain and orbits with contrast and fat suppression showed no abnormalities and NMO/MOG antibody titers were negative. CT angiogram of the neck was found to be normal. As a result, NAION secondary to COVID was diagnosed after an extensive workup which ruled out all other possible causes. At 6-month follow up, the BCVA had improved to 6/6 in both eyes, and the contrast had improved to 1.95 in both eyes; however, there was superior sectoral pallor in the RE as shown in Figure 1c,d and corresponding thinning of the superior retinal nerve fiber layer (RNFL) and the ganglion cell complex in the optical coherence tomography (OCT) as shown in Figure 1e. The inferior altitudinal field defect in the RE persisted.

### 2.2. Case 2

**Figure 2:**
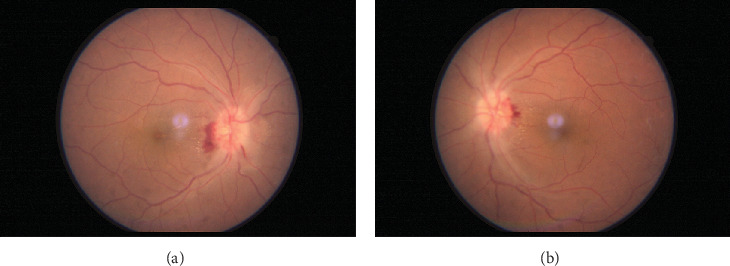
(a, b) Color fundus photograph of the right and left eyes showing bilateral established disk edema with a hyperemic and elevated optic nerve head with blurring of disk margins and peripapillary hemorrhages and hard exudates.

A 41-year-old female admitted for fever after testing positive for COVID-19 for 3 days complained of bilateral sudden diminution of vision for 1 day associated with headache and vomiting. She had no systemic comorbidities except hyperthyroidism. She did not endorse the use of any hormonal medications, including oral contraceptives. On ophthalmology consultation, she had bilateral BCVA of 6/12 and sluggishly reacting pupils in both eyes. Other anterior segment examinations were within the normal range. Dilated fundus examination showed established disk edema with splinter hemorrhages and tortuous vessels in both eyes, as shown in Figure 2a,b. Color vision was normal, but she had bilateral decreased contrast sensitivity (1.65 log unit). She had a constricted peripheral visual field with an enlarged blind spot on testing of the visual fields. Optical coherence tomography–retinal nerve fiber layer analysis (OCT-RNFL) showed disk edema with a thickened RNFL.

Blood investigations include HbA1c, liver function test, renal function test, thyroid function test, lipid profile, ESR, ANA, VDRL, and CRP within normal limits (Table 1). MRI of the brain and magnetic resonance venography (MRV) showed dural venous sinus (both transverse and sagittal) thrombosis. Thrombotic profile (Protein C, Protein S, Antithrombin III, lupus anticoagulant, b2 glycoprotein and anticardiolipin antibodies, and homocysteine) was also normal. Hence, the diagnosis of papilledema secondary to COVID-19 associated cerebral venous sinus thrombosis (CVST) was established. The patient was evaluated by the neuromedicine team, and treatment was started with injection enoxaparin 60 mg twice/day for 15 days, followed by tablet dabigatran 150 mg once/day and acetazolamide 500 mg twice/day. At 3 months, her visual acuity improved to 6/9 bilaterally, and disk edema resolved with ensuing pallor of the optic disk.

### 2.3. Case 3

A 40-year-old male was referred for ophthalmological consultation after he complained of inward deviation of the RE associated with diplopia. He had been admitted for COVID-19-associated pneumonia 1 month back. After 10 days of admission, he developed thrombosis of the left common iliac artery for which he had to undergo above-knee amputation of the left leg. He was a Type II diabetic patient for the past 10 years. He had no other systemic illnesses.

**Figure 3:**
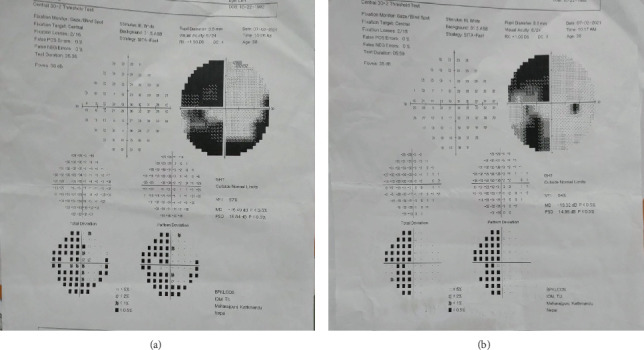
(a, b) Humphrey visual field analysis (30-2) of the left eye and the right eyes showing a left-sided homonymous hemianopia.

On ocular examination, he had BCVA of 6/6 in both eyes. Examination of the extraocular motility showed restriction in the right lateral gaze. Pupillary examination and the rest of the anterior and posterior segment findings were within normal range. He had normal color vision but slightly decreased contrast sensitivity (1.80 log units in the RE and 1.75 log units in the LE) as tested on the Pelli–Robson chart. The Hess chart showed underaction of the right lateral rectus (RLR) and secondary inhibition palsy of the left lateral rectus muscle (LLR). The AVF 30-2 report showed left-sided homonymous hemianopsia as shown in Figure 3a,b. His blood investigation findings are reported in Table 1. MRI of the brain revealed findings suggestive of infarction involving the right occipital lobe and posterior limb of the right internal capsule.

His treatment was continued on tablet aspirin (150 mg), tablet clopidogrel (75 mg), and tablet pregabalin (75 mg) from the neurology side. In the subsequent visit at 3 months, his lateral rectus palsy and diplopia had resolved with full ocular motility in all cardinal gazes; however, his visual fields remained unchanged.

## 3. Discussion

Neurologic manifestations of COVID-19 have been described since the beginning of the pandemic, with the incidence ranging from 36% [5] to more than 50% [6] in various studies. Although various neuro-ophthalmologic manifestations of COVID-19 infection have been reported, the exact incidence is unknown, and our knowledge is built on case reports. In this article, we report three cases of COVID-19 infection with neuro-ophthalmic manifestations. The mechanisms causing neuro-ophthalmic manifestations are proposed to be due to direct viral invasion, hypoxia, hypercoagulability, inflammatory reactions related to cytokine storm, delayed autoantibody formation, endothelial dysfunction, or retrograde axonal transport of the infection via cranial and peripheral nerves [3, 7, 10].

NAION is caused by occlusion of the short posterior ciliary arteries and presents as unilateral, sudden, painless irreversible vision loss, which typically occurs upon waking. It has a higher prevalence in patients with diabetes, hypertension, and ischemic heart disease, hyperlipidemia, hypercoagulability states, tobacco smokers, and in patients with small cup-to- disk ratios [16]. Our first case did not have any vascular comorbidities like diabetes and hypertension, hyperlipidemia but did have an anatomically crowded disk and had a COVID-19 infection. Infection with COVID may have led to a severe inflammatory response with hypercoagulability and hypoxemia and probably decreased vascular compliance in a crowded disk leading to NAION. Arteritic ischemic optic neuropathy (AION) is a mandatory differential diagnosis to rule out in a patient presenting with ischemic optic neuropathy. The difference between AION and NAION in the funduscopic examination is the pallor of the optic nerve head in the AION in contrast to the hyperemic and swollen one in the NAION, along with laboratory tests of elevated ESR and CRP in AION [17]. Our patient did not have any clinical signs or laboratory features compatible with AION or giant cell arteritis, thus ruling out the diagnosis.

The presence of a hypercoagulable state and evidence for the development of thrombotic complications in patients with COVID-19 was gathered early on in the COVID pandemic and even treatment guidelines for thromboembolism in COVID-19 had been published by the World Health Organization (WHO) [2, 18–20]. The mechanism for hypercoagulability could be due to severe inflammatory response, viral endothelitis, blood viscosity, and complement-mediated microangiopathy. The hypercoagulability induced by COVID-19 could have a role in our first case with NAION, resulted in the development of dural sinus thrombosis in our second case, and caused cerebrovascular accidents and deep vein thrombosis in our last case. There are numerous other case reports where patients with COVID-19 developed CVST and papilledema and variable vision loss [21–27]. Our second patient developed CVST and mild respiratory symptoms simultaneously. She did not have any identifiable predisposing factors for CVST other than COVID-19; however, it is still debatable whether COVID-19 was contributing or causal factor in the development of CVST.

Our last case, a 40-year-old male, had right sixth cranial nerve palsy, ischemic stroke involving the right occipital lobe and posterior limb of the right internal capsule, along with thrombosis of the left common iliac artery with diabetes, again proving that hypercoagulability secondary to COVID-19 may be the contributing factor. Various other case reports in the literature have reported isolated cranial nerve palsy, with most commonly sixth nerve involvement, as in our case [12, 13]. There are several other case reports of patients with visual impairment and blindness due to occipital ischemic stroke [28–30]. Most of the cases reported were either elderly and/or had other vasculopathic risk factors like hypertension, diabetes, and SLE. Our patient was a relatively young male with diabetes and multiple site involvement, indicating severe disease.

## 4. Conclusion

Although novel coronavirus (SARS-CoV-2) mainly causes severe acute respiratory syndrome with manifestation seen in the lungs, it can affect multiple organ systems and cause various neuro-ophthalmic manifestations. Neuro-ophthalmic manifestations in our series caused mild to moderate vision loss, probably due to a severe inflammatory response to COVID-19, leading to hypercoagulability, thrombosis, hypoxemia, and endotheliopathy.

## Figures and Tables

**Table 1:** Summary of blood investigation findings in the three cases.

	**Case1: NAION**	**Case 2: Papilledema associated cerebral venous sinus thrombosis**	**Case 3: Acute ischemic stroke with right lateral rectus palsy and left homonymous hemianopsia**
HbA1c	5.5%	5.9%	7.1%
Liver function test	WNL	WNL	WNL
Renal function test	WNL	WNL	WNL
Thyroid function test	WNL	WNL	WNL
Lipid profile	WNL	WNL	WNL
Erythrocyte sedimentation rate (ESR)	9	15	19
C- reactive protein (CRP)	1.05 (N)	1.85 (N)	4.0(N)
ANA	Negative	Negative	Negative
RA	Negative	Negative	Negative
VDRL	Nonreactive	Nonreactive	Nonreactive
Serology HIV	Nonreactive	Nonreactive	Nonreactive
Serology HBsAg	Nonreactive	Nonreactive	Nonreactive
PT/INR	14	12	15
Coagulation profile	WNL	WNL	Not done
Sleep study	N	Not done	Not done
MRI brain and orbit/MRV	N	Transverse and sagittal sinus thrombosis	Infarction involving the right occipital lobe and posterior limb of the right internal capsule

## Data Availability

Data sharing is not applicable to this article as no new data were created or analyzed in this study.
